# Sternoclavicular Joint Reconstruction Using a Double Figure-of-8 Technique With Palmaris Longus Autograft Augmented by an Internal Brace: The Criss-Cross Technique

**DOI:** 10.1016/j.eats.2025.103693

**Published:** 2025-06-18

**Authors:** Blaise Pellegrini, Xavier Lannes, Aurelien Traverso, Filippo Gerber, Patrick Goetti

**Affiliations:** a Department of Orthopedics and Traumatology, Lausanne University Hospital and University of Lausanne, Lausanne, Switzerland; b Service of Orthopedics and Traumatology, Ensemble Hospitalier de la Côte, Morges, Switzerland

## Abstract

Sternoclavicular joint (SCJ) dislocation is a rare injury that typically occurs following high-energy trauma. Posterior dislocation can lead to major injuries such as vascular, tracheal, and esophageal compression with potentially life-threatening issues. Reconstruction of the SCJ is indicated in locked posterior dislocation, failed conservative management with persistent symptomatic instability, and/or secondary scapular dyskinesia. Numerous techniques have been described to stabilize the SCJ. One of the most popular reconstruction techniques is the figure-of-8 using an autograft, usually a semitendinosus or gracilis. Here, we present a double figure-of-8 reconstruction technique using oblique bicortical bone tunnels with an ipsilateral palmaris longus autograft augmented by an internal brace. This technique provides a strong reconstruction and allows an ipsilateral upper limb graft harvest.

Sternoclavicular joint (SCJ) dislocations (Fig 1) are rare injuries, but they can be life-threatening in cases of posterior dislocation due to the proximity of mediastinal structures.^1^ Regarding acute cases, SCJ stabilization is indicated for locked posterior dislocation or recurrent instability following closed reduction. Chronic posterior SCJ dislocations are frequently underdiagnosed at initial presentation and should be treated to prevent complications such as subclavian vein compression or tracheoesophageal fistula.^2^ In contrast, surgical reconstruction for chronic anterior dislocations should be reserved for patients with persistent symptoms such as SCJ pain or scapular dyskinesia that is recalcitrant to appropriate conservative treatment.^3^ Several surgical techniques for the treatment of acute and chronic SCJ instability have been described in the literature.^4^ One of the most popular techniques is a figure-of-8 reconstruction with tendon autografts, using the sternocleidomastoid muscle, palmaris longus (PL) tendon, semitendinosus or gracilis tendon, with or without augmentation with synthetic braids, anchor sutures, or temporary plating.^5^ A biomechanical study by Spencer and Kuhn^6^ showed improved restoration of SCJ stability, particularly in the posterior direction with bicortical figure-of-8 tendon reconstruction, compared to monocortical intramedullary bone tunnels and subclavius reconstruction techniques. Lacheta et al.^7^ reported a survivorship of 90% for this construct at a 5-year follow-up. We present a modified double figure-of-8 SCJ reconstruction using a PL autograft, allowing a double-crossing construct augmented with a 2-mm FiberTape (Arthrex) internal brace, which we believe to be a more reliable construct compared to the graft alone.

**Fig 1:**
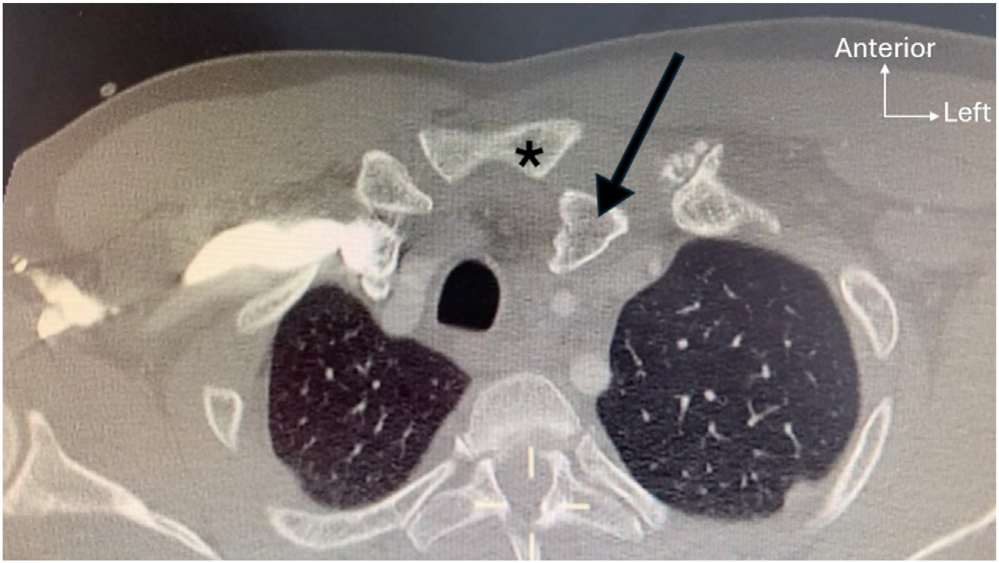
Axial computed tomography scan revealing a locked posterior dislocation of the left sternoclavicular joint. Medial left clavicle (black arrow); manubrium (asterisks).

## Surgical Technique

The surgery procedure is shown in Video 1; the main steps are described in the following section. Pearls and pitfalls are highlighted in Table 1.

**Table 1:** Pearls and Pitfalls of Double Figure-of-8 SCJ Reconstruction

Pearls	Pitfalls
Patient positioning: ▪ Place a bump between the shoulder blades to assist in SCJ reduction. ▪ Drape the entire thorax in case of emergency thoracotomy.	Preoperative planning: ▪ Performing Schaeffer’s test is mandatory. Absence of a palmaris longus tendon may result in the inadvertent harvest of an incorrect structure and/or lead to intraoperative delays with the need for alternative grafts.
Graft harvest: ▪ Use a 2-incision graft harvest technique with a closed tendon stripper to maximize graft length.	Graft harvest: ▪ Carefully inspect potential graft damage after harvest or insufficient length before proceeding as it may compromise the reconstruction.
SCJ exposure: ▪ Preserve the sternocleidomastoid insertion for better postoperative clavicular stability. ▪ Perform subperiosteal dissection of the medial clavicle and manubrium to reduce the risk of major vessel lesion.	
Tunnel drilling: ▪ Start with a 2.5-mm drill bit to guide the 4.5-mm holes, ensuring correct trajectory and avoiding posterior wall blowout.	Tunnel drilling: ▪ Use retractors and drill under direct visualization when possible, as misaligned or too posteriorly drilled tunnels can cause injury to mediastinal structures. ▪ Undertaking this procedure in a facility without available vascular surgical support may endanger the patient, as there is a potential risk of injury to major vascular structures. ▪ The obliquity of the tunnels should not exceed 45°, as a greater angle increases the risk of failing to perforate the posterior cortex, potentially compromising the construct’s stability.
Graft and FiberTape passage: ▪ Use shuttle relays to ensure smooth graft passage and reduce iatrogenic graft damage. ▪ Graft and FiberTape (Arthrex) must be properly tensioned at each hole passage to avoid loose tension in the final construct.	Graft and FiberTape passage: ▪ Improper tensioning at each step can result in asymmetric forces, leading to persistent instability.
Fixation and final tensioning: ▪ Secure FiberTape before graft knotting to ensure optimal reduction control. ▪ Finally, reinforce graft fixation with additional FiberWire sutures for backup fixation.	Rehabilitation: ▪ Strict adherence to rehab protocol is essential, as premature strengthening or range of motion can lead to construct failure or potential graft elongation.

SCJ, sternoclavicular joint.

### Patient Positioning

**Fig 2:**
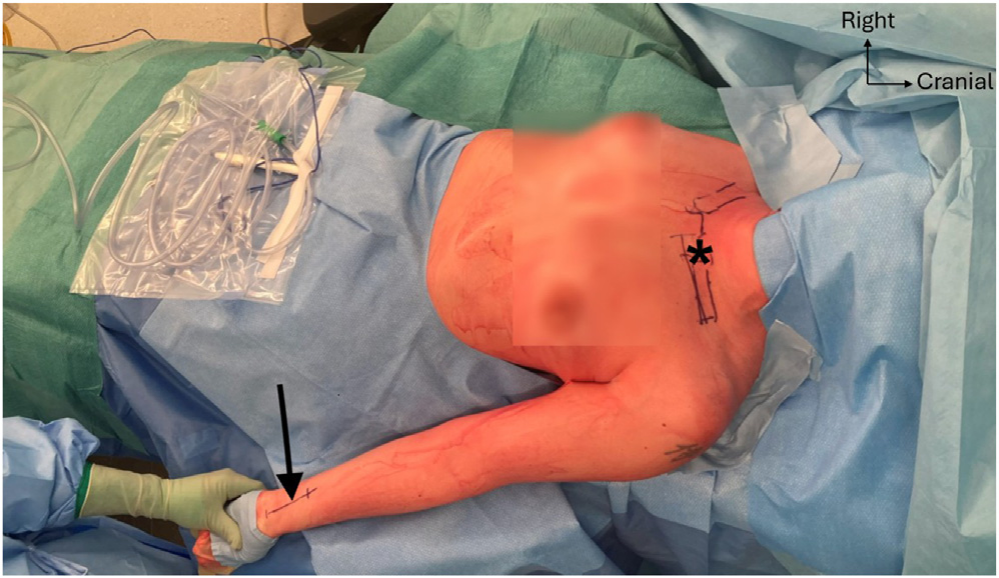
The patient is placed in a supine position with a bump positioned between both shoulder blades to facilitate sternoclavicular joint (SCJ) exposure and reduction. The entire thorax and arm on the injured left side are draped for the palmaris longus (PL) graft harvest. Site of PL graft harvest (black arrow); dislocated SCJ (asterisks).

The patient is placed in a supine position with a bump positioned between both shoulder blades to facilitate SCJ reduction. The entire thorax and arm on the injured side are draped for the PL graft harvest (Fig 2). Note that access to the entire thorax is desirable in case of perioperative major vessel injury, necessitating an emergency thoracotomy.

### Palmaris Longus Graft

**Fig 3:**
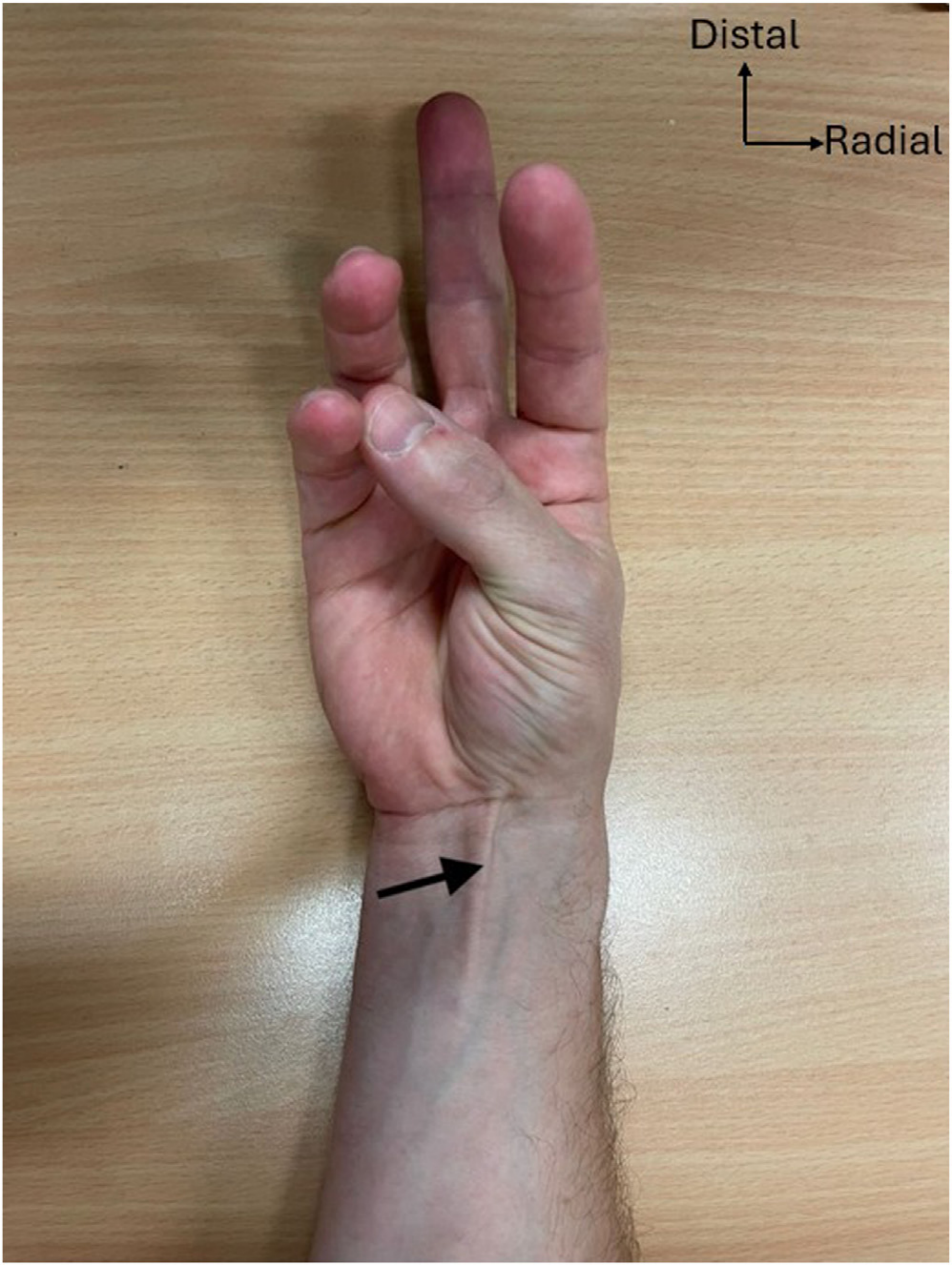
Preoperative Schaeffer’s test. Active opposition of the thumb against the fifth finger test demonstrated on a right hand showing the presence of the palmaris longus tendon (black arrow) preoperatively.^8^

**Fig 4:**
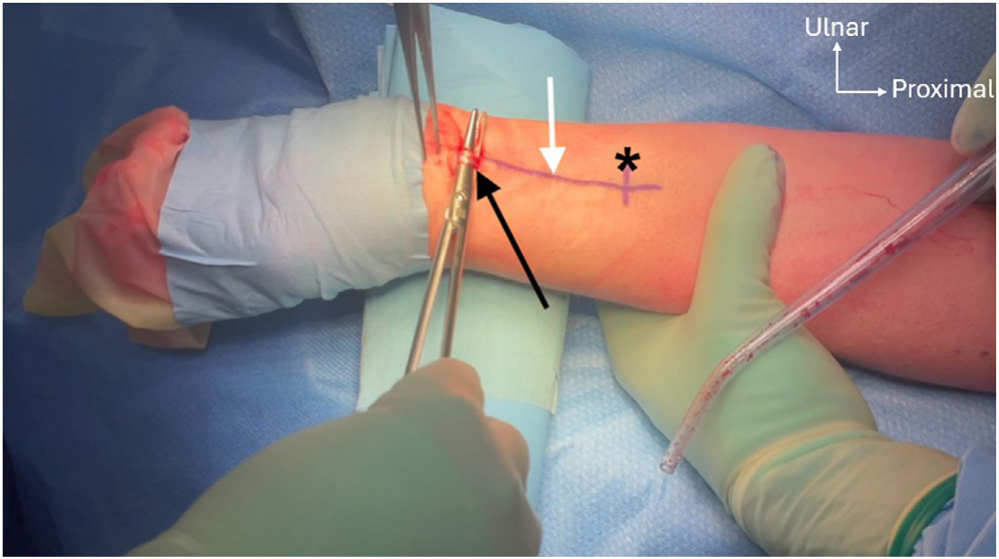
Palmaris longus (PL) tendon harvest. The left PL tendon (black arrow) is exposed through a small transverse incision at the level of the wrist flexion crease. Before the incision, the course of the tendon is palpated and marked (white arrow), as well as the second skin incision 8 cm more proximally (asterisks).

**Fig 5:**
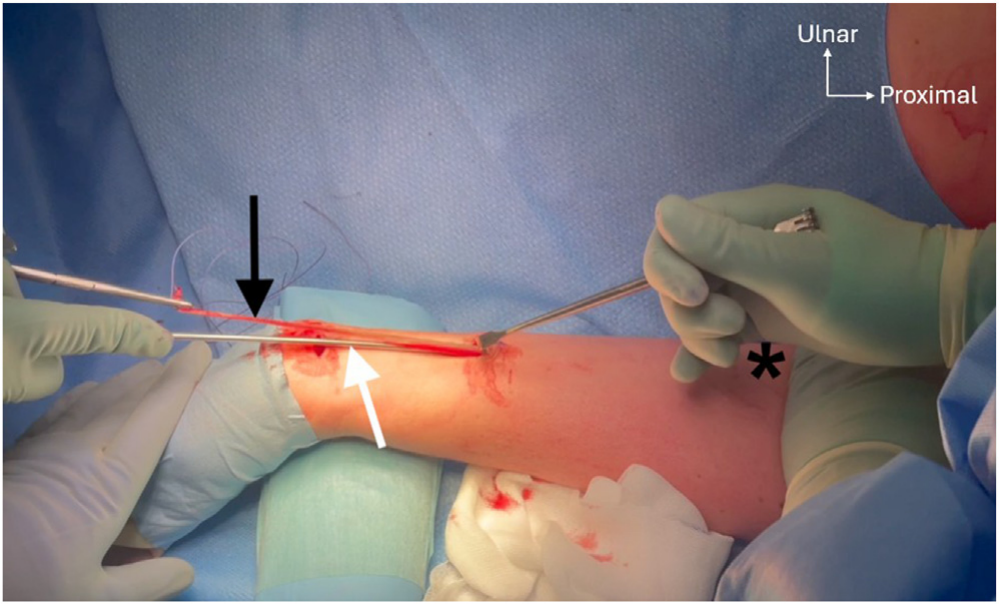
Harvest of the left palmaris longus tendon (black arrow) using a double-incision technique, and a closed tendon stripper (white arrow) directed toward the flexor pronator mass (asterisks) to optimize graft length.

**Fig 6:**
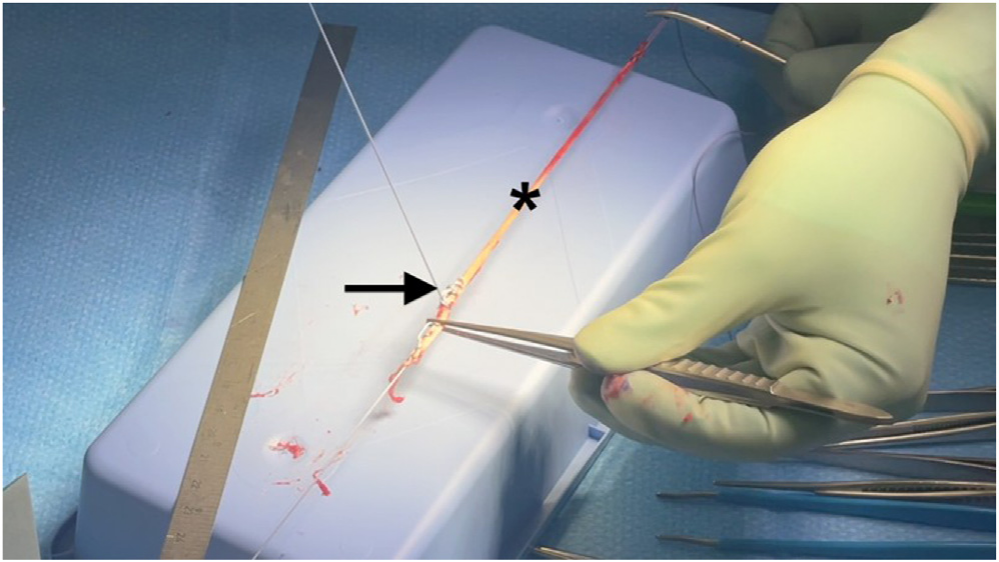
The left palmaris longus graft (asterisks) is secured and reinforced using a nonabsorbable No. 2 FiberWire (Arthrex) suture at both ends with a Krakow suture technique (black arrow).

Preoperative confirmation of the presence of the PL is conducted by active opposition of the thumb against the fifth finger (Schaeffer’s test)^8^ (Fig 3). First, the PL graft is harvested using a 2-incision technique. A transverse incision of about 1.5 cm is made at the level of the wrist flexion creases. After subcutaneous dissection, the PL tendon is identified, exposed, and prepared (Fig 4). A traction suture is placed to allow proximal tendon preparation with scissors, followed by PL tenotomy distally to the traction suture and then introduction of the graft into a closed tendon stripper. The tendon stripper is stopped approximately 8 to 10 cm proximally to the first incision, and another small transverse incision is made to optimize maximal graft length during stripping. The tendon is externalized from this second incision and then extracted, using the traction suture or a nonsharp clamp, with the closed tendon stripper directed to the flexor-pronator mass (Fig 5). After inspection of the graft, it is then secured with a reinforced nonabsorbable suture at both ends using a Krakow suture technique with a No. 2 FiberWire (Arthrex) (Fig 6).

### Sternoclavicular Joint Exposure

**Fig 7:**
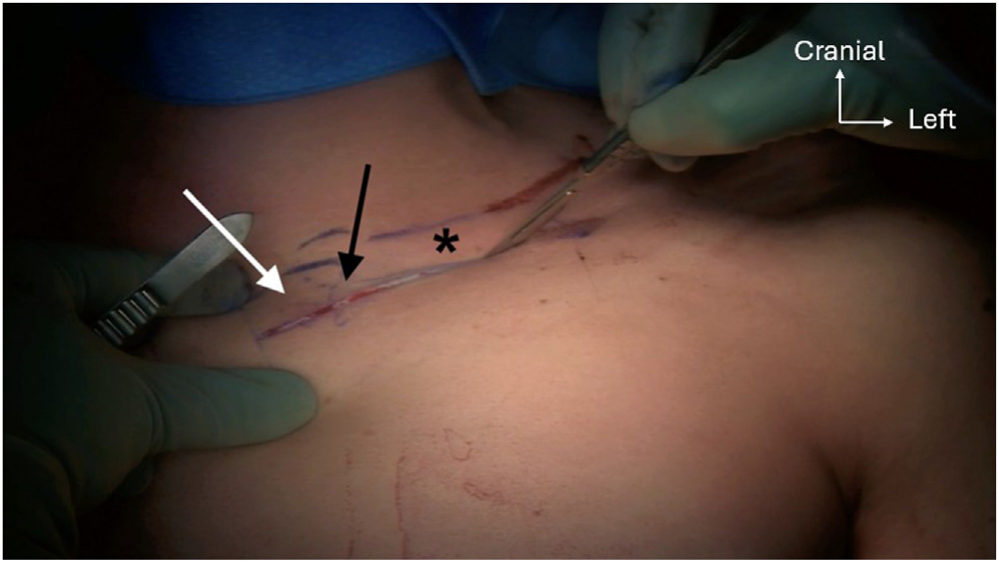
A 6- to 8-cm skin incision is made in front of the left sternoclavicular joint (black arrow). Medial clavicle (asterisks) and manubrium (white arrow).

**Fig 8:**
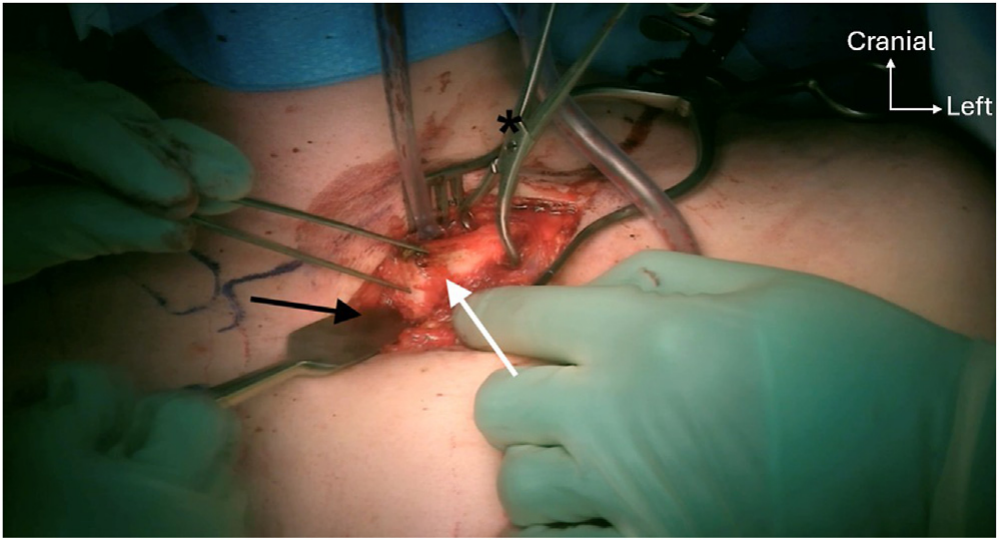
A pointed clamp (asterisks) is placed on the left medial clavicle (white arrow) to reduce the sternoclavicular joint. A large bone elevator (black arrow) is placed posterior to the clavicle to protect the posterior structures ahead of bone drilling.

A 6- to 8-cm skin incision is made in front of the SCJ (Fig 7). After subcutaneous dissection, the SCJ capsule is opened and a traction clamp is placed on the clavicle, allowing easier subperiosteal preparation of the medial edge of the clavicle and manubrium (Fig 8), preserving the insertion of the sternocleidomastoid muscle. The sternoclavicular disk is removed from the joint. The medial clavicle and manubrium are prepared subperiosteally using a raspatory. Great care must be taken when dissecting posterior to the joint regarding the underlying vessels.

### Sternoclavicular Joint Fixation

**Fig 9:**
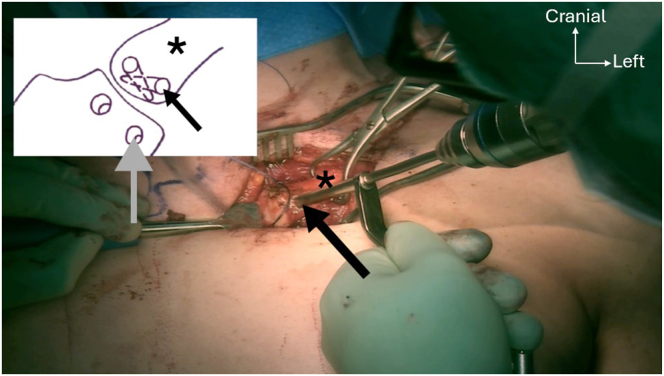
The inferior oblique clavicular distal tunnel (black arrow) is drilled from anterodistal to posteroproximal, taking care not to cross the superior oblique tunnel. Left clavicle is marked with an asterisk. Note that both manubrium tunnels (gray arrow) are drilled in a strict anteroposterior plane, perpendicular to the surface of the manubrium.

**Fig 10:**
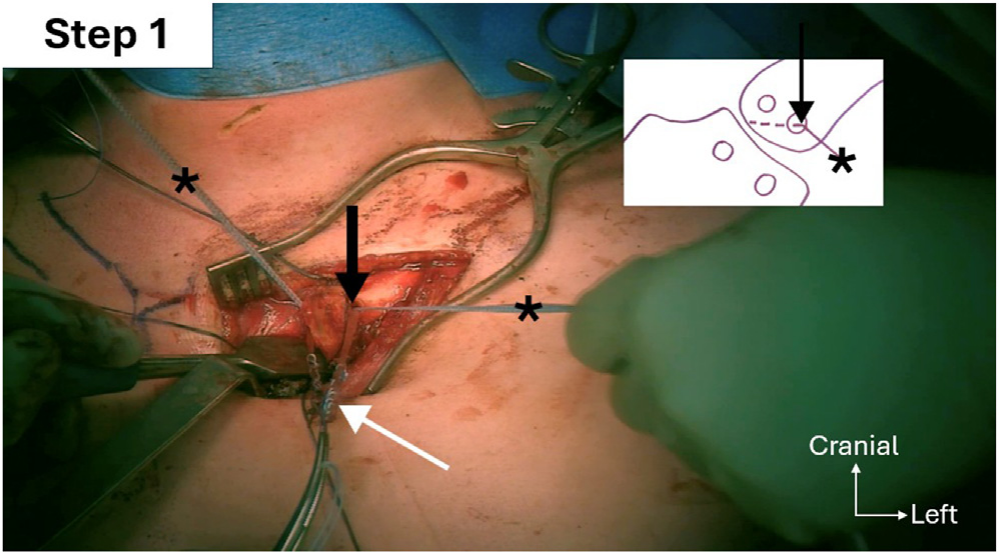
Step 1 of double figure-of-8 passage sequence: passage of FiberTape (Arthrex; asterisks) through the distal left clavicle oblique tunnel (black arrow), from anteroinferior to posterosuperior. The palmaris longus autograft (white arrow) follows the same course.

**Fig 11:**
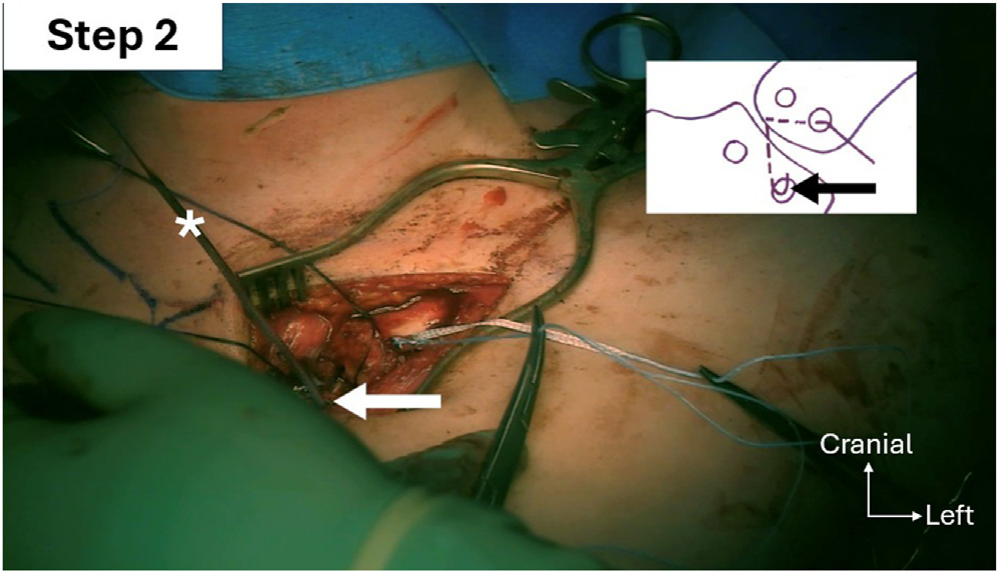
Step 2 of double figure-of-8 passage sequence: passage of the graft and the FiberTape (Arthrex; white asterisks) into the manubrial distal inferior tunnel (black and white arrows) from posterior to anterior (left sternoclavicular joint).

**Fig 12:**
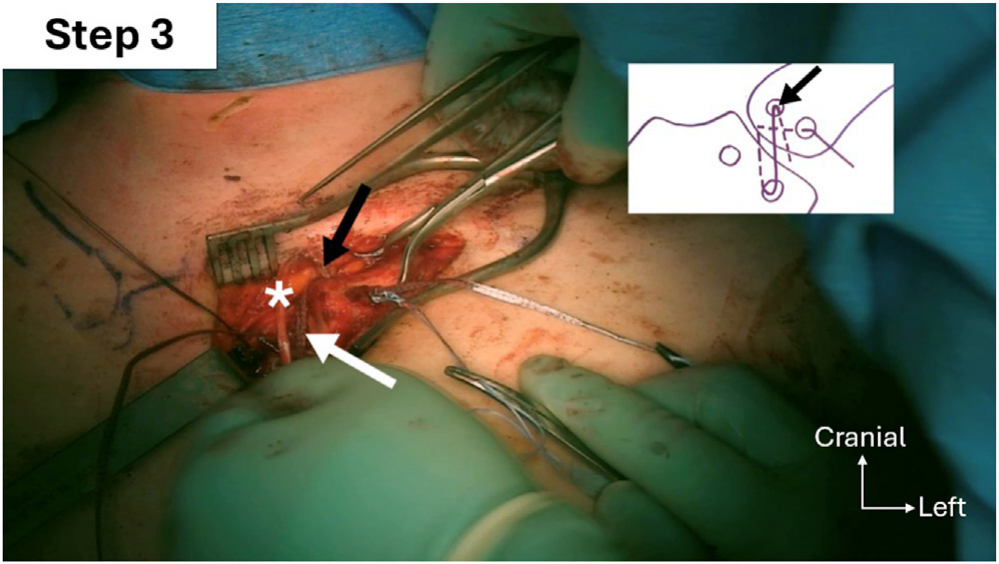
Step 3 of double figure-of-8 passage sequence: passage of the graft (white asterisks) and FiberTape (Arthrex; white arrow) into the left clavicular anteroproximal oblique tunnel from anteroproximal (black arrow) to posterodistal.

**Fig 13:**
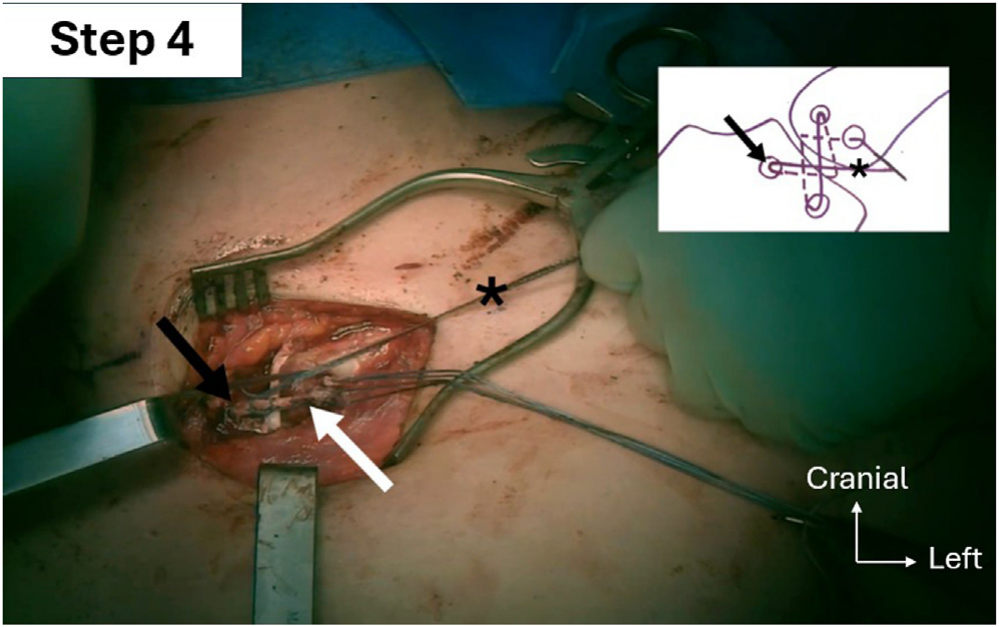
Step 4 of double figure-of-8 passage sequence: graft (white arrow) and FiberTape (Arthrex; asterisks) passage into the manubrial proximal tunnel, entering posterior and exiting anterior (black arrow), before crossing in the direction of the left clavicle anterodistal tunnel.

**Fig 14:**
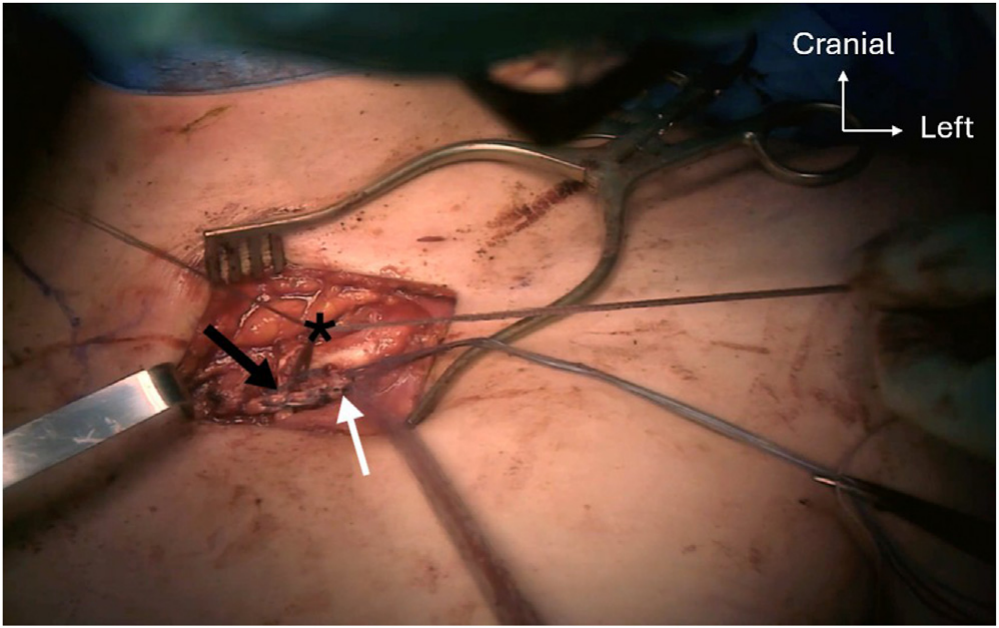
After left sternoclavicular joint reduction, the FiberTape (Arthrex) internal brace is tensioned and knotted first (asterisks). Both ends of the palmaris longus tendon autograft (white arrow) are knotted and secured with tendon-to-tendon FiberWire (Arthrex) sutures at the level of the sternoclavicular joint (black arrow).

Two 4.5-mm anteroposterior holes are drilled into the proximal and distal parts of the manubrium, starting with a 2.5-mm drill bit to ensure correct tunnel placement, which is then over-reamed using a 4.5-mm drill bit. Following the same technique, an oblique 4.5-mm anteroproximal clavicle hole is drilled directed to the posterodistal part of the clavicle. This is followed by a second oblique 4.5-mm anterodistal clavicle hole, directed to the posterior-proximal part (Fig 9). Shuttle relays with No. 2 Vicryl (Ethicon) are placed in each hole with a Hewson suture passer. The PL autograft and a 2-mm FiberTape (Arthrex) are passed into the clavicular anterodistal hole using the preplaced shuttle relay, from anterior to posterior (Fig 10). The graft and the FiberTape are then passed into the manubrial distal hole using the previously placed shuttle relay, from posterior to anterior (Fig 11). The graft and the FiberTape are then passed into the clavicular anteroproximal oblique hole from anterior to posterior (Fig 12). Care must be taken to tension the graft and the FiberTape at each hole passage, to ensure adequate final tensioning of the construct. Finally, the graft and the FiberTape are passed into the manubrial proximal hole from posterior to anterior. The SCJ is reduced, and then the tendon graft and FiberTape are tensioned to ensure good final stability (Fig 13). The FiberTape is secured first (Fig 14). After adequate tensioning, the graft is then itself secured with knotting of both ends. The graft knots are protected with multiple nonresorbable No. 2 FiberWire sutures between both ends of the tendon into the knots as additional off-loading and protection sutures.

### Closing

**Fig 15:**
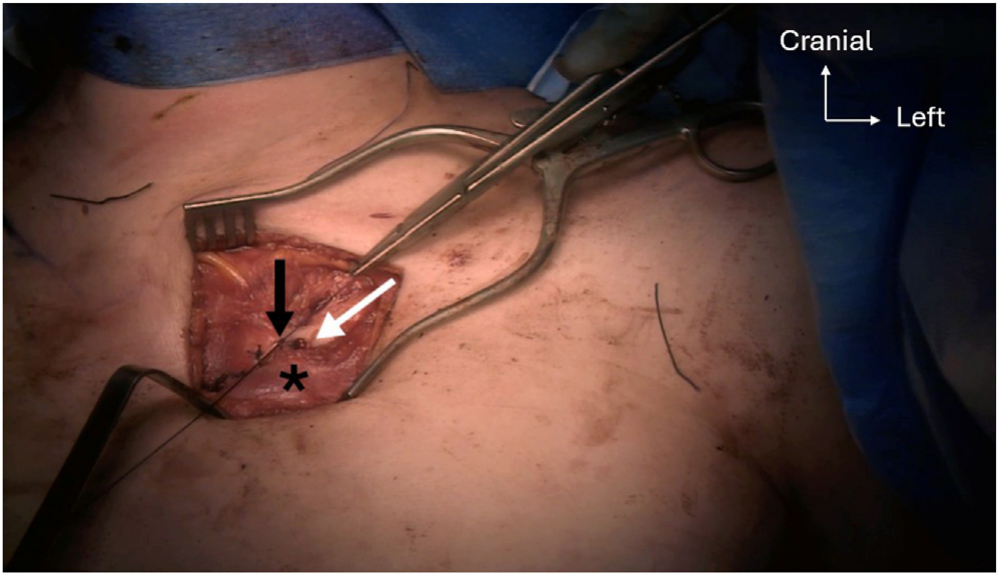
Left sternoclavicular joint capsule (asterisks) and fascia overlying the medial clavicle (white arrow) are closed using No. 0 Vicryl (Ethicon; black arrow) to ensure good coverage and additional stability of the construct.

After irrigation and disinfection, the SCJ capsule and fascia over the joint and medial clavicle are closed using No. 0 Vicryl (Ethicon) to ensure good covering and additional stability of the construct (Fig 15). Skin is closed with a running intradermal locked suture using 3/0 PDS (Johnson & Johnson).

### Postoperative Rehabilitation

The affected shoulder is placed in a sling for 4 weeks with gentle passive range of motion during this period. From 5 to 11 weeks postoperatively, active assisted and active range of motion are encouraged. After 12 weeks, strengthening with weightlifting is allowed.

## Discussion

The key benefit of our technique is the enhanced primary stability provided by augmenting the PL autograft with a FiberTape internal brace. The double figure-of-8 construct also acts as a self-centering force compared to the classic simple figure-of-8 technique, thereby avoiding anterior or posterior SCJ subluxation. Several tendons are described as autografts for figure-of-8 constructs in SCJ reconstruction, including the sternocleidomastoid muscle, semitendinosus, gracilis, and tibialis anterior tendons. The advantages of PL autografts include harvesting using the same draping, which is convenient and time efficient, and low donor site morbidity (1%) without a functional impact on grip and wrist flexion when compared to potential deficits in knee flexion strength and anterior knee pain reported with semitendinosus and gracilis autografts.^9^ A biomechanical study performed by Solon et al.^10^ found that PL and gracilis tendons show similar mechanical properties and microstructural alignment under load. The length of the PL tendon is adequate for SCJ reconstruction, with a mean length of 123.6 mm for men and 111.4 mm for women.^11^ In addition, the smooth surface of the tendon makes it easy to pass through the transosseous tunnels, and it exhibits a good stiffness of 42 N/m^2^, making it adequate for articular stabilization.^12^ The use of an autograft is further favored over that of an allograft to reduce the risk of infection, as well as maximize healing and long-term tissue integration. Studies have shown a higher failure rate in ligament reconstruction performed with allografts.^13^ The downside of this technique lies in PL absence in around 15% of the population,^14^ which can be identified with Schaeffer’s test.^8^ Concerning the obliquity of the holes in SCJ reconstruction, a biomechanical study conducted by Martetschläger et al.^15^ showed that oblique holes lead to a less robust construct compared to straight holes. This assertion should not be extended to our technique. In fact, this study included holes with obliquity in the lateral-medial direction to reduce the risk to the posterior structures, with the drill exit located on the medial border of the sternum rather than the posterior side. In our technique, holes are drilled with a cranial/caudal obliquity and stay bicortical in the anteroposterior plane. Injuries to vessels and mediastinal structures are prevented using a raspatory during manubrium and medial clavicle posterior subperiosteal preparation, as well as hole drilling. Potential disadvantages of this technique are the less intuitive sequence of graft passage, which necessitates strict adherence to the surgical protocol to avoid construct failure, along with the need for a longer graft to accommodate the double figure-of-8 configuration. Nonetheless, in our experience, PL grafts provide adequate length to achieve a stable and reliable construct.

The double figure-of-8 technique using a PL autograft, augmented with a FiberTape internal brace, is in our experience a valid technique for SCJ stabilization (Table 2).

**Table 2:** Advantages and Disadvantages of Sternoclavicular Joint Reconstruction Using a Double Figure-of-8 Technique With Palmaris Longus Autograft Augmented by an Internal Brace

Advantages	Disadvantages
▪ Time efficient due to ipsilateral arm draping	▪ Palmaris longus absence in 15% of the population
▪ Low donor site morbidity	▪ The sequence of graft passage is technically demanding and necessitates strict adherence to the surgical protocol to avoid construct failure
▪ Potential increased stability with a bicortical double figure-of-8 reconstruction	▪ Additional cost of FiberTape (Arthrex) internal brace
▪ Enhanced primary stability with FiberTape internal brace	▪ Need for a longer graft to accommodate the double figure-of-8 configuration compared to a standard figure-of-8 technique

## Disclosures

All authors (B.P., X.L., A.T., F.G., P.G.) declare that they have no known competing financial interests or personal relationships that could have appeared to influence the work reported in this paper.

## Supplementary Data

Video 1 Sternoclavicular joint reconstruction using a double figure-of-8 technique with palmaris longus (PL) autograft augmented by an internal brace. The patient is placed in a supine position. The entire thorax and arm on the injured side (left) are draped for the PL graft harvest. Preoperative confirmation of the presence of the PL is conducted by active opposition of the thumb against the fifth finger. First, the PL graft is harvested using a 2-incision technique and then secured with a reinforced nonabsorbable suture at both ends using a Krakow suture technique with a No. 2 FiberWire (Arthrex). After subcutaneous dissection, the left sternoclavicular joint capsule is opened. The medial clavicle and manubrium are prepared subperiosteally using a raspatory. Two 4.5-mm anteroposterior holes are drilled into the proximal and distal parts of the manubrium, followed by 2 oblique holes drilled in the medial part of the clavicle. The PL autograft and a 2-mm FiberTape (Arthrex) are passed through the tunnels in a double figure-of-8 configuration and then secured by tying knots between the 2 ends of each. The sternoclavicular joint capsule and fascia over the joint and medial clavicle are closed to ensure additional stability. Finally, skin is closed with a running intradermal locked suture.
